# Positive Attitudes towards Non-Invasive Prenatal Testing (NIPT) in a Swedish Cohort of 1,003 Pregnant Women

**DOI:** 10.1371/journal.pone.0156088

**Published:** 2016-05-19

**Authors:** Ellika Sahlin, Magnus Nordenskjöld, Peter Gustavsson, Josephine Wincent, Susanne Georgsson, Erik Iwarsson

**Affiliations:** 1 Department of Molecular Medicine and Surgery and Center for Molecular Medicine, Karolinska Institutet, CMM L8:02, Karolinska University Hospital, S-171 76, Stockholm, Sweden; 2 Sophiahemmet University, Stockholm, Sweden; 3 Department of Clinical Science, Intervention and Technology, Karolinska Institutet, Stockholm, Sweden; Hospital Authority, CHINA

## Abstract

**Objective:**

The clinical utilization of non-invasive prenatal testing (NIPT) for identification of fetal aneuploidies is expanding worldwide. The aim of this study was to gain an increased understanding of pregnant women’s awareness, attitudes, preferences for risk information and decision-making concerning prenatal examinations with emphasis on NIPT, before its introduction into Swedish healthcare.

**Method:**

Pregnant women were recruited to fill in a questionnaire, including multiple-choice questions and Likert scales, at nine maternity clinics located in different areas of Stockholm, Sweden.

**Results:**

In total, 1,003 women participated in the study (86% consent rate). The vast majority (90.7%) considered examinations aiming to detect fetal abnormalities to be good. Regarding NIPT, 59.8% stated that they had heard about the method previously, yet 74.0% would like to use the test if available. The main factor affecting the women’s decision to undergo prenatal chromosomal screening was worry about the baby’s health (82.5%), followed by the urge to have as much information as possible about the fetus (54.5%). Most women (79.9%) preferred to receive NIPT information orally.

**Conclusion:**

The overwhelming majority of a cohort of 1,003 pregnant women considered prenatal examinations good. Moreover, the majority had a positive attitude towards NIPT and would like to use the test if available.

## Introduction

Non-invasive prenatal testing (NIPT) for identification of chromosomal aneuploidies is the most recent addition to the growing palette of fetal examination methods available today. The test is possible because cell-free fetal DNA is present in maternal blood during pregnancy [1]. A fetal trisomy will give rise to a small gain in DNA fragments from the trisomic chromosome, which can be detected with high accuracy using massive parallel sequencing [2–7]. The sensitivity and specificity as well as the negative predictive value of the method is >99% for trisomy 21 (Down syndrome), with slightly lower performance for trisomy 13 and 18 [8–11]. However, the positive predictive value varies depending on the prevalence of trisomy in the population being tested, and is not higher than 50–80% in an unselected pregnant population [8,9,12]. As a consequence, NIPT is not considered a diagnostic test, and invasive testing by amniocentesis or chorionic villus sampling (CVS) is therefore recommended to confirm the presence of a chromosomal abnormality in case of a positive test result [13]. Although recent studies indicate that the invasive tests are very safe [14,15], they have traditionally been burdened with a non-negligible risk of miscarriage [16,17]. Additionally, the invasive procedure *per se* is often experienced as unpleasant, as well as it requires fetal medicine experts to perform the test. Therefore, the invasive tests have never been an option for large-scale testing, and screening methods have been developed to identify women at high risk of fetal aneuploidy. As NIPT has a much higher accuracy compared with current other screening methods, many invasive tests could be avoided if NIPT was used as the primary screening [12]. Still, questions have arisen regarding how to best implement NIPT into clinical practice. Previous studies have shown positive attitudes towards NIPT among both the public and pregnant women [18–21]. However, as the test only requires a blood sample, there are concerns that it will be taken without sufficient consideration [22,23], that its accuracy in combination with the simplicity of the test may lead to normalization of selective abortions [24], and that people with physical and intellectual disabilities might face an increased stigmatization in society [25,26]. It is of great importance to provide expectant parents with accurate information concerning prenatal examinations, associated risks, and potential consequences of a positive test result. However, this task might be challenging for health professionals as risk information is highly complex [27], and additionally, risk perception varies depending on factors such as personal background, gender and socio-economic status, and is highly influenced by emotions and affect [28].

This study was performed in Stockholm before, but in close proximity to, the introduction of NIPT into Swedish healthcare. At the time of data collection, pregnant women in Stockholm were offered the first trimester combined test (FCT), and 64% took the test (data from 2014) [29]. However, NIPT was available at a few private clinics at a cost of approximately €1,000 and NIPT ads could be found regularly in local newspapers. The aim was to gain an increased understanding of pregnant women’s awareness, attitudes, preferences about risk information and decision-making concerning prenatal examinations with emphasis on NIPT, as well as to gain knowledge about how this new method would be accepted by the potential users. Additionally, attitudes towards having a child with a chromosomal abnormality were studied, as well as the women’s self-perceived likelihood for that event to occur. A large cohort of 1,003 pregnant women was included to obtain a substantial material that could serve as valuable input when presenting information prior to prenatal examinations.

## Methods

### The questionnaire

The questionnaire used for data collection was study specific and developed by the research group. The group, which was interdisciplinary, consisted of a biomedical scientist, a consultant and two senior consultants in Clinical Genetics as well as a midwife experienced in questionnaire development within this research area. The questionnaire included background questions (age, mother tongue, educational level, gestational week, parity, previous miscarriages) as well as multiple choice questions and Likert scales covering attitudes, knowledge, preferences for risk information and decision-making regarding prenatal examinations, with emphasis on NIPT. One question regarding attitudes towards prenatal examinations had been used in previous studies [30]. The questionnaire also covered attitudes towards having a child with a chromosomal abnormality, and the self-perceived likelihood of that event to occur. The questionnaire was available in Swedish, designed to be quick and easy to fill in, and took approximately five minutes to complete.

### Study participants and procedure

Pregnant women in any week of gestation were recruited to participate in the study. They were recruited in waiting rooms of nine different maternity clinics located in different areas of Stockholm, selected to represent a broad socioeconomic variety of the participants. All participants were provided oral and written information about the study, whereupon they filled in the questionnaire directly in the waiting room. The questionnaire was filled in anonymously, and all participants had the possibility to withdraw from the study by not handing in the questionnaire. To understand women’s current awareness and spontaneous attitudes, no background information was given regarding the prenatal examination methods covered in the questions. However, the researchers were available to answer questions at all times during the completion of the questionnaires. The study was approved by the Regional Ethical Review Board in Stockholm (DNR 2014/1817-31/4). All data were collected between January 27 and June 3, 2015, i.e. before the introduction of NIPT into Swedish healthcare.

### Statistical analysis

All calculations were performed using the IBM Statistical Package for the Social Sciences (SPSS) version 22. Descriptive statistics were used to extract answer frequencies to the questions in the questionnaire. Fisher’s exact test was used to assess statistically significant differences in answer proportions between subgroups in case of binary answer alternatives, and the Chi2 test was used in case of multiple answer alternatives. The significance level was set to p<0.05. Five-point Likert scales were compressed to two or three categories to avoid small cell sizes.

## Results and Discussion

### The participants

In total, 1,163 women were asked to participate in the study. The consent rate was 86%, resulting in 1,003 participants (Table 1). The main reason why women declined participation was because of language difficulties (59%), followed by lack of interest (20%) or lack of time (17%). Some participants did not answer all the questions and thus the sample size varies slightly by question.

**Table 1:** Characteristics of 1,003 pregnant women recruited to answer a questionnaire about prenatal examinations.

Participant characteristics (n = 1,003)	
**Place of recruitment, n (%)**	
Stockholm city center (2 districts)	437 (43.6%)
suburban areas (7 districts)	566 (56.4%)
**Age, years**	
mean ± SD	31.6 ± 5
median	32
[range]	[16–52]
missing values	6
**Gestational age, weeks**	
mean ± SD	25.9 ± 9.8
median	28
[range]	[6–42]
missing values	16
**Number of born children, n (%)**	
0	435 (44.3%)
1–2	511 (52.0%)
3–4	31 (3.1%)
≥5	5 (0.5%)
missing values	21
**Number of miscarriages, n (%)**	
0	655 (66.7%)
1–2	239 (24.3%)
3–4	33 (3.4%)
≥5	4 (0.4%)
missing values	72
**Mother tongue, n (%)**	
Swedish	763 (78.5%)
Arabic	25 (2.5%)
Spanish	17 (1.6%)
Polish	14 (1.4%)
other (60 languages represented)	153 (15.7%)
missing values	31
**Education, n (%)**	
university, ≥2 years	680 (67.8%)
high-school	264 (26.3%)
elementary school	36 (3.6%)
other	22 (2.2%)
missing values	1
**Performed fetal examinations, n (%)**	
**YES**	844 (84.1%)
ultrasound	662 (78.4%)
FCT	557 (66%)
amniocentesis	21 (2.5%)
CVS	39 (4.6%)
other	29 (3.4%)
- NIPT	9 (1%)
missing values	0

FCT = first trimester combined test, CVS = chorionic villus sampling, NIPT = non-invasive prenatal testing.

### Attitudes towards prenatal examinations

The vast majority of the participants considered examinations aiming to detect fetal abnormalities to be good. On a five-point Likert scale where 1 = “Good” and 5 = “Bad”, 90.7% scored 1–2. Only 1.4% scored 4–5, and 7.9% scored 3, i.e. “Neither good nor bad”. A majority of the women (68.8%) perceived prenatal examinations as calming, and 56.2% stated that it was self-evident to undergo such examinations. However, one fourth (26.8%) perceived prenatal examinations as frightening (Table 2). When the women were asked about their attitudes towards specific prenatal examination methods, 96.9% stated that they were positive towards prenatal ultrasound, whereas the corresponding proportion regarding amniocentesis/CVS was only 42.1%. Regarding FCT and NIPT, the corresponding proportions were similar (78.0% vs. 73.0%, respectively) (Table 3). This is in line with the knowledge that women prefer examinations without risk for the fetus [31]. However, the positive attitude towards NIPT, as well as for FCT in this cohort did not reach the level of ultrasound examination. This may be explained by that ultrasound is appealing also to women who, for various reasons, do not want to perform prenatal chromosomal screening. Furthermore, to view the baby on the screen is a very strong emotional experience for the expectant parents, and is even a facilitator for attachment [32]. Košek *et al*. recently developed a measure of anxiety due to prenatal diagnostic procedures. They showed that women having an amniocentesis had a higher procedure-induced anxiety than women having an ultrasound examination. However, the women experienced the same level of fear of having an abnormal result [33]. Regarding NIPT, the procedure-induced anxiety will likely be lower than for amniocentesis as it is not associated with any risk for the fetus, but probably higher than for ultrasound examination as having to wait for the result is an anxiety-inducing factor [33].

**Table 2:** Pregnant women’s attitudes towards prenatal examinations.

I think that examinations aiming to detect fetal abnormalities are … (%)			
**Good**	**Neither**	**Bad**	
90.7	7.9	1.4	Total n = 974
**Frightening**	**Neither**	**Not frightening**	
26.8	28.2	45.1	Total n = 845
**Not calming**	**Neither**	**Calming**	
9.8	21.5	68.8	Total n = 848
**Not self-evident**	**Neither**	**Self-evident**	
18.3	25.6	56.2	Total n = 839

**Table 3:** Pregnant women’s attitudes towards specific prenatal examination methods.

What is your attitude towards … (%)	Positive	Neither	Negative	Not familiar with the method
**Ultrasound** (n = 955)	96.9	1.8	0.8	0.4
**FCT** (n = 972)	78.0	12.6	5.7	3.7
**Amniocentesis/CVS** (n = 925)	42.1	28.1	15.8	14.1
**NIPT** (n = 934)	73.0	10.0	4.5	12.5

FCT = first trimester combined test, CVS = chorionic villus sampling, NIPT = non-invasive prenatal testing.

### Awareness and interest regarding NIPT

One of the main purposes of this study was to gain an understanding of how NIPT would be received by Swedish pregnant women as the method was soon to be introduced into Swedish healthcare. In this cohort, 588 out of 983 women (59.8%) stated that they had heard about NIPT previously. The women were asked if they would like to use NIPT if available. On a five-point Likert scale where 5 = “Yes, I am completely sure” and 1 = “No, absolutely not”, 74.0% of the women (n = 723) claimed that they would like to use it, 12.1% women (n = 118) stated that they would not use it, and 13.9% (n = 136) did not know. It is striking that women who had never heard about NIPT stated that they would like to do the test just by hearing about it in the questionnaire. This suggests that the short description, “it is possible to take a blood sample in early pregnancy that with high accuracy can tell if the fetus has a chromosomal abnormality”, which was the only information provided, is very appealing. Previous studies have shown that pregnant women’s interest in NIPT differs greatly between countries, ranging from 51% in the Netherlands [34] to 88% in the UK [20]. The results of the present study are similar to a Californian study including 114 pregnant women, where 71.9% were interested in using NIPT [21].

The women were asked whether they would need information to facilitate their decision about whether or not to undergo NIPT, and although most of the women already seemed to have decided one way or the other, a great majority stated that they would need more information. Of those who stated that they wanted to have the test, 83.5% (n = 599) requested information, whereas the corresponding proportion was significantly lower, 64.1% (n = 75), among the women who did not want the test (p<0.001). This may indicate that women who are against chromosomal screening are more firm in their belief. Not surprisingly, almost all women (91.0%, n = 122) who were uncertain whether they would like to use NIPT stated that they would need information. By far the most requested alternative regarding how to receive information was orally by the midwife, selected by 638 of the 801 women who requested information (79.7%). This puts high demands on the midwives in terms of genetic knowledge and counseling skills, e.g. to describe the relationship between sensitivity/specificity and positive predictive value in an apprehensible fashion, to avoid that women perceive NIPT as a diagnostic test [35]. However the women could choose more than one alternative, and many (n = 392, 48.9%) selected oral in combination with written information. A separate appointment with a doctor or midwife was less requested (n = 187, 23.3%) as was information on the internet (n = 167, 20.8%).

### Desired information and willingness to pay regarding NIPT

The women were asked what information they would like from a NIPT analysis, and could score either “Yes” or “No” for each alternative provided. Out of the 859 women who potentially would like to use NIPT, i.e. those who stated that they wanted the test together with the women who were not sure, half (50.4%) stated that they would like to know the fetal sex, 96.3% would like to know if the fetus had Down syndrome, and 98.0% would like to know if the fetus had another, more severe chromosomal abnormality. Additionally, 93.2% would like to receive information about all detectable chromosomal abnormalities (Table 4). A large, recent study of women and health professionals in nine different countries showed that women in general placed the greatest emphasis on comprehensive information and test safety, whereas health professionals placed more emphasis on test accuracy and that the test could be performed in early pregnancy [36]. Similar results were shown in a Dutch study including 507 pregnant women, where the participants were willing to accept a far less accurate test to obtain more information about the fetal chromosome status [37].

**Table 4:** Pregnant women’s information preferences after undergoing NIPT.

What information would you like to receive after undergoing NIPT?	Women who would potentially undergo NIPT		Women who would *not* undergo NIPT		P-value (Fisher’s exact test)
	Total n	YES (%)	Total n	YES (%)	
The fetal sex	701	50.4	101	40.6	0.079
If the fetus has Down syndrome	781	96.3	98	48.0	<0.001
If the fetus has another, more severe chromosomal abnormality	787	98.0	103	68.9	<0.001
All chromosomal abnormalities that are detectable	809	93.2	98	50.0	<0.001

Conversely, out of the women in our cohort who would not use NIPT, only 48.0% would like to know if their fetus had Down syndrome. However, considerably more (68.9%) would like to know if the fetus had another, more severe chromosomal abnormality (Table 4). There is an ongoing debate regarding if it is ethically defensible to screen for Down syndrome. In a questionnaire study including 284 people with Down syndrome living in the United States, almost all participants reported that they live happy and fulfilling lives, and that they share similar hopes and dreams as people without the syndrome [38]. However, in a study including mothers of children with Down syndrome, the majority thought NIPT to be a good thing; mainly because detection in early pregnancy would allow for time to prepare to care for a child with the condition [26].

Out of the 859 women that potentially would like to undergo NIPT, 78.1% (n = 671) stated that they would be willing to pay for themselves if NIPT was not covered by the national health insurance. However, most of them (n = 498, 74.2%) were willing to pay €50 to €100, i.e. the lowest price range of the alternatives given, and considerably less than the cost of a commercial NIPT analysis. Approximately one fifth (n = 147, 21.9%) would pay between €200 and €2,000, and a small number were willing to pay as much as €5,000 (n = 5, 0.9%). In a Dutch study, the mean price that participants were willing to pay for NIPT was €169, i.e. close to the €150 that women ≤36 years pay for FCT in the Netherlands [39]. In Sweden, healthcare is generally free of charge, which probably affects the willingness to pay for specific analyses.

### Attitudes towards chromosomal abnormalities and self-perceived risk

Almost one third (n = 306, 30.5%) of all women in the cohort stated that it would not matter if their baby was born with a chromosomal abnormality such as Down syndrome, although a number of women wrote comments such as, “I would be sad about the fact that the child would not be healthy, but I would not terminate a pregnancy because of it”, or “it would probably be hard at first, but I would love the child just as much anyway”. Conversely, 44.9% (n = 450) stated that they would react negatively, and 17.9% (n = 180) would find it very negative. No statistical differences were identified between nulli- and multiparas (p = 0.777), or between women who had experienced miscarriages or not (p = 0.382). However, women willing to do NIPT stated that they would react negatively or very negatively to a significantly higher extent than women who would not like to do the test (76.7% vs. 36.3%, p<0.001).

**Fig 1:**
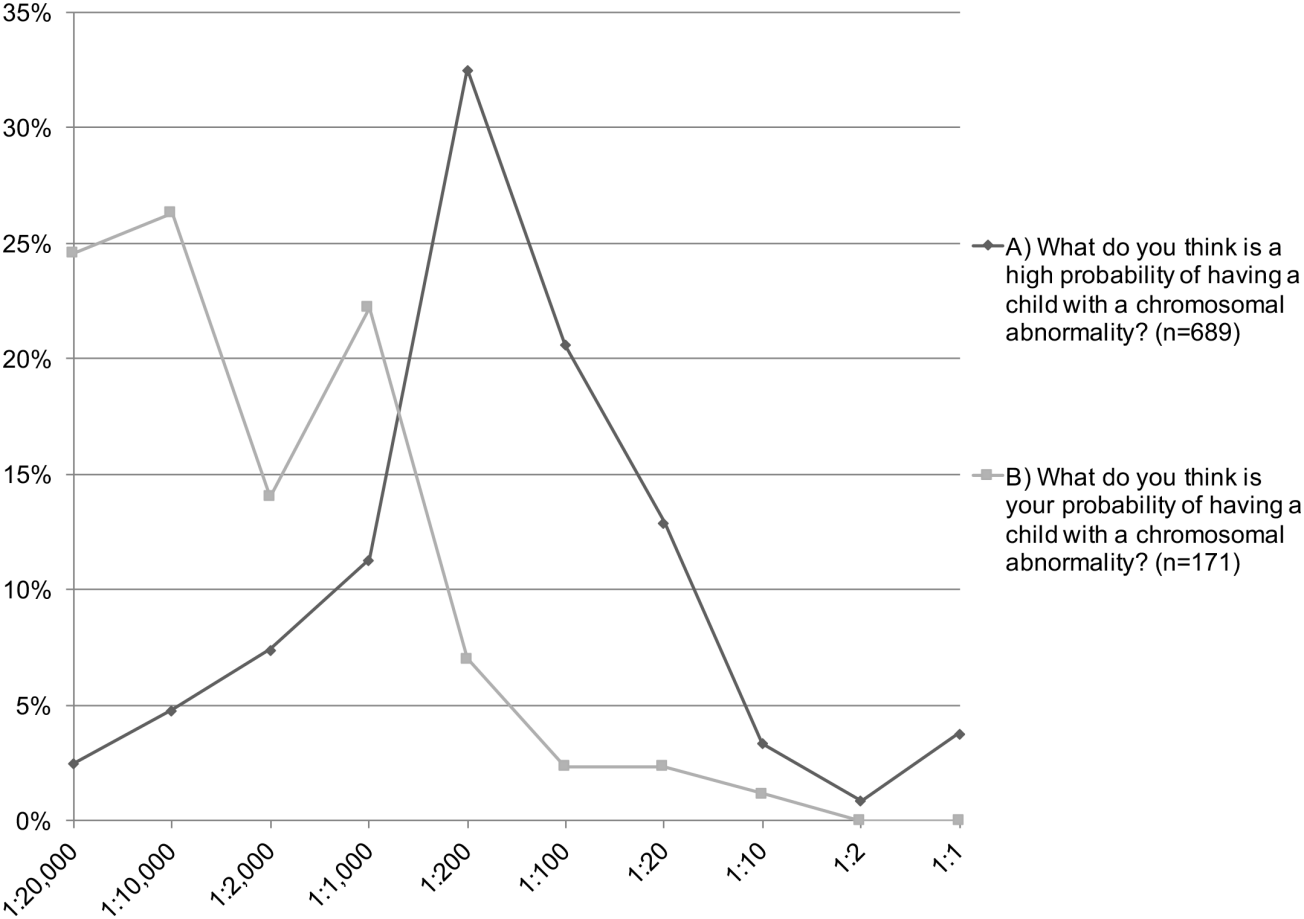
Proportion of pregnant women’s perception of what they consider a high probability of having a child with a chromosomal abnormality (A) and the perception of their risk of having a child with a chromosomal abnormality (B). The women answered the question by selecting one of the answers displayed on the x-axis. In B, women who had performed the first trimester combined test or invasive testing were excluded.

The women were asked about their self-perceived likelihood of having a child with a chromosomal abnormality. On a five-point Likert scale ranging from “Not likely at all” to “Very likely”, 72.8% stated that they thought it was unlikely, whereas 3.6% thought it was likely, and 20.8% stated that they did not know. The results did not differ between women below or above 35 years of age (p = 0.618). The women were asked to specify what they thought was a high probability by selecting an alternative on a scale ranging from 1:1 to 1:20,000. The most frequent answer, selected by 32.5% (n = 224) was 1:200, i.e. the threshold used as high risk in FCT screening in Sweden. However, almost one third (n = 301, 30.4%) answered, “I don’t know”, indicating that they either did not know, or that they did not know how to interpret the values. The challenge of communicating risk has been addressed in previous studies [40], and the development of tools to facilitate this issue has been requested [41]. The women were also asked to specify what they thought was their own probability of having a child with a chromosomal abnormality. After excluding women who had gone through FCT, amniocentesis or CVS (as they would already be aware of their probability), there were 419 women who potentially could have given an answer. However, seven values were missing, and as many as 241 women (57.5%) answered “I don’t know”. Out of the 171 women giving an answer, 50.9% (n = 87) thought their probability was 1:20,000 or 1:10,000. The distribution of all answers is displayed in Fig 1.

### Decision making regarding prenatal chromosomal screening

The main factor affecting the women’s decision to undergo prenatal chromosomal screening was worry about the baby’s health (n = 799, 82.5%), followed by the urge to have as much information as possible about the fetus (n = 528, 54.5%). Only a small proportion stated that they were affected by expectations from others (n = 25, 2.6%) or the feeling that “everyone else is having such tests” (n = 5, 0.5%). The women could also state another, own option, and 3.5% (n = 35) wrote, “mental preparation” (Table 5). Out of the 118 women who stated that they did not want to use NIPT, 20.3% (n = 24) wrote that nothing influenced their decision as they were already convinced that they did not want to do such tests, and another 38.1% did not choose any of the given alternatives, which further strengthens the hypothesis that the women who not want to perform prenatal chromosomal screening are more convinced about their choice. This is in line with a study that showed that women declining prenatal screening make informed decisions to a higher extent than screening acceptors [30], which could partially be explained by that many women perceive prenatal screenings as routine analyses [42].

**Table 5:** Pregnant women’s opinions about what affects their decision to undergo chromosomal screening on their fetus.

What affects your decision to undergo chromosomal screening on your fetus? n = 968	%
Worry about the baby's health	82.5
I want to know as much as possible	54.5
I do not see any reason to decline	26.3
Own experience by person with a chromosomal abnormality or other severe congenital disease	15.8
The values of society	7.1
It is important to know the fetal sex	3.2
Expectations from others	2.6
Everyone else is having such tests	0.5
**Other**	12.2
- Mental preparation	3.5
- Nothing, I do not want to do such tests	2.4
- Worry about the life-change in having a disabled child/effects on the family	1.0

When asked about who influences the women’s decision to undergo prenatal chromosomal screening, 95.8% (n = 932) stated that it is their own decision. However, the women could select more than one alternative, and the majority (n = 660, 67.9%) selected themselves together with their partner. Considerably less women stated that they were affected by the midwife or doctor at the maternity clinic (n = 137, 14.1% vs. n = 98, 10.1%, respectively). Hopefully, this reflects that expectant parents feel sufficiently informed to make their own choice and that the health professionals have managed to deliver information about the analyses in an objective manner.

### Study strengths and limitations

This study includes a large number of participants who had access to equal maternity care conditions. All data were collected before NIPT was available via national healthcare. The maternity clinics were selected to represent a wide variety in socioeconomic status of the participating women. A limitation of the study is that the questionnaire was only available in Swedish. Although a few non-Swedish speaking women were able to complete the questionnaire with the help of an interpreter, 93 women declined participation because of language difficulties. This was especially a problem in the suburban areas. The study reflects opinions of well-educated pregnant women living in a larger city and can not be generalized to the pregnant population as a whole. However, the large cohort may reflect the views of women living under similar circumstances.

## Conclusion

The overwhelming majority of a cohort of 1,003 pregnant women considered examinations aiming to detect fetal abnormalities to be good. Moreover, the majority was positive towards NIPT and would like to use the test if available.

## Supporting Information

S1 Dataset (XLSX)

S1 Questionnaire English translation of the questionnaire used in the study. (PDF)
