# Supplementary material for: Positive Attitudes towards Non-Invasive Prenatal Testing (NIPT) in a Swedish Cohort of 1,003 Pregnant Women
Source: PLoS One. 2016 May 19;11(5):e0156088. doi: 10.1371/journal.pone.0156088 (PMC4872994; doi:10.1371/journal.pone.0156088)
Supplement: S1 Questionnaire — (PDF) [file pone.0156088.s002.pdf]

Maternity clinic: ....
Date: ....

Questionnaire about prenatal examinations

Age: .... **Gestational week:** ....

Number of children: .... **Previous miscarriages:** ....

Mother tongue: ....

Education:

☐ Elementary school or similar
☐ High school or similar
☐ University, two years or more
☐ Other: ....

1. Have any examinations been performed on your fetus? ☐ Yes ☐ No

If yes, **which?**

☐ First trimester combined test (FCT)
☐ Amniocentesis
☐ Chorionic villus sampling
☐ Ultrasound examination, week: ....
☐ Other: ....

2. I think that examinations aiming to detect fetal abnormalities are … *(Score one alternative per row.)*

a) Good	☐	☐	☐	☐	☐	Bad
b) Frightening	☐	☐	☐	☐	☐	Not frightening
c) Not calming	☐	☐	☐	☐	☐	Calming
d) Not self-evident	☐	☐	☐	☐	☐	Self-evident

**3. What is your attitude towards:**	**Very positive**				**Very negative**	**Not familiar with the method**
First trimester combined test (FCT)	☐	☐	☐	☐	☐	☐
Amniocentesis/chorionic villus sampling	☐	☐	☐	☐	☐	☐
Ultrasound	☐	☐	☐	☐	☐	☐
Blood sample från the mother which with high accuracy can tell if the fetus has a chromosomal aberration so-called non-invasive prenatal testing (NIPT)	☐	☐	☐	☐	☐	☐

4. How would you react upon having a child with a chromosomal aberration (such as Down syndrome)?

☐ It would not matter
☐ Negatively
☐ Very negatively

5. How likely do you think it is that your expected child has a chromosomal aberration?

Very likely ☐ ☐ ☐ ☐ ☐ Not likely at all

6a. What do you think is a high probability of having a child with a chromosomal aberration?

☐1:20 000 ☐1:10 000 ☐1:2000 ☐1:1000 ☐1:200 ☐1:100 ☐1:20 ☐1:10 ☐1:2 ☐1:1

☐ I don’t know

6b. What do you think is *your* probability of having a child with a chromosomal aberration?

☐1:20 000 ☐1:10 000 ☐1:2000 ☐1:1000 ☐1:200 ☐1:100 ☐1:20 ☐1:10 ☐1:2 ☐1:1

☐ I don’t know

7a. Have you heard that it is possible to take a blood sample in early pregnancy that with high accuracy can tell if the fetus has a chromosomal aberration?

☐ Yes ☐ No

7b. Would you like to have such a test if it was available?

Yes, I am completely sure ☐ ☐ ☐ ☐ ☐ No, absolutely not

7c. Would you need information to help your decision if to perform such a test? ☐ Yes ☐ No

If yes, **how would you like to receive the information?** *You may select several answers.*

☐ Oral information by the midwife at the maternity clinic
☐ A separate visit to a doctor/midwife
☐ Written information
☐ Information on the internet
☐ Other: ………………………………………………………………

7d. If this blood sample would not be covered by the national health insurance, would you be willing to pay by yourself? ☐ Yes ☐ No

If yes, **how much would you be willing to pay?**

☐ €50 ☐ €100 ☐ €200 ☐ €500 ☐ €1000 ☐ €5000

**8. What information would you like to get after undergoing NIPT?**	**Yes**	**No**
The fetal sex	☐	☐
If the fetus has Down syndrome	☐	☐
If the fetus has another, more severe chromosomal abnormality	☐	☐
All chromosomal abnormalities that are detectable	☐	☐

9a. What affects your decision to undergo chromosomal examinations on your fetus? *You may select several answers.*

☐ Expectations from others
☐ Worry about the baby's health
☐ The values of the society
☐ I do not see any reason to decline
☐ It is important to know the fetal sex
☐ Everyone else is having such tests
☐ I want to know as much as possible
☐ Own experience by person with a chromosomal abnormality or other severe congenital disease
☐ Other: ………………………………………………………………

9b. Who affects your decision to undergo chromosomal examinations on your fetus? *You may select several answers.*

☐ Myself
☐ My partner
☐ Family and friends
☐ The midwife at the maternity clinic
☐ The doctor at the maternity clinic
☐ Other: ………………………………………………………………

Thank you for your participation!
